# A Low-Measurement-Cost-Based Multi-Strategy Hyperspectral Image Classification Scheme

**DOI:** 10.3390/s24206647

**Published:** 2024-10-15

**Authors:** Yu Bai, Dongmin Liu, Lili Zhang, Haoqi Wu

**Affiliations:** Electronic and Information Engineering, Shenyang Aerospace University, Shenyang 110136, China

**Keywords:** hyperspectral image (HSI) classification, small-sample, triplet network classifier (TNC), feature mixture based active learning (FMAL), dual-strategy pseudo-active learning (DSPAL)

## Abstract

The cost of hyperspectral image (HSI) classification primarily stems from the annotation of image pixels. In real-world classification scenarios, the measurement and annotation process is both time-consuming and labor-intensive. Therefore, reducing the number of labeled pixels while maintaining classification accuracy is a key research focus in HSI classification. This paper introduces a multi-strategy triple network classifier (MSTNC) to address the issue of limited labeled data in HSI classification by improving learning strategies. First, we use the contrast learning strategy to design a lightweight triple network classifier (TNC) with low sample dependence. Due to the construction of triple sample pairs, the number of labeled samples can be increased, which is beneficial for extracting intra-class and inter-class features of pixels. Second, an active learning strategy is used to label the most valuable pixels, improving the quality of the labeled data. To address the difficulty of sampling effectively under extremely limited labeling budgets, we propose a new feature-mixed active learning (FMAL) method to query valuable samples. Fine-tuning is then used to help the MSTNC learn a more comprehensive feature distribution, reducing the model’s dependence on accuracy when querying samples. Therefore, the sample quality is improved. Finally, we propose an innovative dual-threshold pseudo-active learning (DSPAL) strategy, filtering out pseudo-label samples with both high confidence and uncertainty. Extending the training set without increasing the labeling cost further improves the classification accuracy of the model. Extensive experiments are conducted on three benchmark HSI datasets. Across various labeling ratios, the MSTNC outperforms several state-of-the-art methods. In particular, under extreme small-sample conditions (five samples per class), the overall accuracy reaches 82.97% (IP), 87.94% (PU), and 86.57% (WHU).

## 1. Introduction

Hyperspectral images (HSIs) present rich and unique spatial and spectral features in a three-dimensional cubic data structure. As one of the key technologies for observation and exploration in the 21st century, HSI methods have been applied in several crucial fields, such as environmental monitoring [1], land cover evaluation [2], and ocean monitoring [3]. In addition, HSIs also play a significant role in the military field, such as camouflage recognition [4], battlefield space information acquisition [5], differentiation of target and decoy [6], detection of weapons of mass destruction [7], and monitoring of international treaty compliance [8]. HSIs can also play a significant role at different frequency ranges, like X-ray, ultraviolet, visible and near-infrared, and terahertz [9,10,11,12,13].

In these applications, HSI’s multiband spectral resolution provides valuable spectral data support for various industries, significantly improving the accuracy of analysis and decision making. However, the classification of HSIs remains a primary challenge, making HSI classification a continuous research hotspot within the academic community. Especially in the case of limited training samples, classification performance can easily decrease. Meanwhile, the original HSI is often affected by the spectral changes caused by sensor noise and environmental conditions [14,15]. Therefore, how to intelligently construct a streamlined training set with limited training data and effectively handle the large-scale or complex feature space constitutes a key problem.

Earlier studies focused on the spectral features of the pixels, covering support vector machines (SVMs) [16], K-nearest neighbors [17], polynomial logistic regression [18], sparsity algorithms of object recognition [19], etc. However, since ground targets are usually spatially continuous, there is strong spatial correlation between adjacent pixels in hyperspectral images. Fu et al. [20] generated joint spectral–spatial features, while Mu et al. [21] performed feature extraction in the spectral and spatial domains separately with two-branch networks. The spectral and spatial processing in these methods is conducted independently, ignoring the joint dependence of spectral and spatial information. Fu et al. [22] introduced tensor singular spectral analysis (TensorSSA) to extract global and low-rank features of HSI, while Dai et al. [23] proposed a smart-HOSVD method for 3D feature extraction in HSI. These methods have yielded promising results in classifying HSIs with small sample sizes. However, the above methods require manual selection and design of key spectral–spatial features to train the model.

In recent years, the development and application of deep learning have propelled the widespread use of convolutional neural networks (CNNs) in areas such as image classification and semantic segmentation [24,25,26]. Compared to traditional methods, deep learning methods exhibit strong advantages that do not require complex hand-crafted feature engineering or much prior knowledge. Hu et al. [27] were pioneers in using convolutional neural networks for HSI classification. One-dimensional CNNs (1D-CNNs) can perform convolution operations along the spectral dimension, effectively capturing the correlations and features between bands. However, since ground targets are usually spatially continuous, there is a strong spatial correlation between adjacent pixels in hyperspectral images. However, the method only considers spectral information while ignoring the potential role of spatial information.

As research in deep learning progressed, researchers designed and adopted 2D-CNNs [28], which typically excel at capturing spatial variations between pixels. Nevertheless, 2D-CNNs do not fully leverage the abundant spectral information available in HSIs. To overcome this limitation, G. Cheng [29] developed a robust spectral–spatial feature representation by fusing spectral features with deep spatial features. Still, 3D-CNNs are more favored as they utilize 3D convolutional kernels to jointly capture spectral and spatial information. This innovative and effective method promptly garnered interest. The HSI cube data extraction approach, implemented using a 3D neural network, does not necessitate any pre-processing to extract its inherent spectral–spatial characteristics. Paoletti et al. [30] presented two separate CNN architectures: one designed for extracting spatial characteristics and the other for extracting spectral characteristics. Zhong et al. [31] introduced a spectral–spatial residual network (SSRN) for learning spectral–spatial representations and employed supervised 3D deep learning for HSI classification. This approach effectively addresses the issue of accuracy deterioration by utilizing consecutive blocks of spectral and spatial residuals. These methods successfully extracted spatial and spectral characteristics and achieved advancements in HSI classification. However, there are still hurdles in terms of effectively utilizing the information. This is because the spatial resolution and spectral resolution in HSI data are inconsistent, leading to potential inconsistencies in the perception capabilities of the 3D convolutional kernel at different scales, which affects classification performance. To alleviate this issue, Roy et al. [32] introduced a hybrid spectral CNN model called HybridSN. The HybridSN model integrates the advantages of 3D-CNNs and 2D-CNNs, effectively learning spatial and spectral features in hyperspectral images. The 3D-CNN is responsible for the initial extraction of spatial–spectral joint features, while the 2D-CNN further abstracts high-level spatial features. In this way, the classification performance of the hybrid convolutional neural network model is enhanced, and compared to using a 3D-CNN alone, the network complexity is reduced, and classification accuracy is improved. Yu et al. [33] introduced a contrastive GCN (ConGcn) that improves contrast learning performance by incorporating a spectral space prior. This enhances the expressive power of the generated representation and thus improves the classification results. Xue et al. [34] introduced a network architecture for a spectral–spatial Siamese network (S3-Net) involving feeding features of different sizes separately into the network. They employed a weighted loss function to enable the model to learn more features when faced with limited samples. While the above-mentioned methods have achieved excellent results, the imbalance between positive and negative sample pairs can impact both the convergence speed and classification performance of the model. Subsequently, Xue et al. introduced a differentiated-scale restricted graph convolutional network (DSR-GCN) [35], building upon S3-Net, to address the issue of missing pixel features caused by sample imbalance. The accuracy of DSR-GCN was remarkable.

Although these methods have achieved great success in hyperspectral image classification, their accuracy heavily depends on a large number of labeled samples.

For HSI classification, the dataset consists of a single hyperspectral image. A portion of the pixels is used as the training set, while the remaining pixels (excluding background and irrelevant areas) serve as the test set.

Pixel-level labeling of HSI involves complex calibration calculations and substantial manual labor, potentially damaging the original features of land cover during extensive labeling. Labeling specific classes requires expert knowledge for accuracy. Current HSI classification algorithms heavily rely on labeled data, and obtaining sufficient labeled data is costly, significantly limiting practical applications. Thus, researching HSI classification with small samples will greatly reduce application costs, lay the foundation for expanding hyperspectral image technology applications, and have significant practical implications.

Liu et al. [36] proposed a deep convolutional neural network model for classifying hyperspectral images with few samples. The training strategy of the model is to gradually increase the number of samples in each class by 5 samples at a time, with the total number of samples ranging from 5 to 25. This approach aims to enhance the learning capability of the model with small batches of data to address the classification challenges in the case of a limited number of samples. Sun et al. [37] introduced an adversarial representation module to extract spectral and spatial features, replacing feature fusion approaches with class consistency. Zhang et al. [38] proposed graph information aggregation cross-domain few-shot learning (Gia-CFSL) to solve the problems of differences in spatial and spectral resolutions of different sensors and differences in the same land cover category. It performs FSL and domain alignment under the condition that all labeled samples are available in the source data and a few labeled samples are available in the target data. These methods consider the issue of HSI small-sample classification from different perspectives and propose various solutions to address the problem.

In the case of small-sample classification, contrastive learning [39] and active learning [40] are considered by scholars to be important methods for improving classification accuracy. Common contrastive learning methods include Siamese networks [41], triplet networks [42], SimCLR [43] (simple framework for contrastive learning of visual representations), and MoCo [44] (momentum contrast). Zhao et al. [45] developed a two-branch concatenated network with common parameters to acquire knowledge about the distinctions among various attributes. Cao et al. [46] introduced a contrast learning approach for a 3D convolutional Siamese network to address the challenge of HSI classification when there is a lack of appropriate data. By integrating contrast learning with conventional label-based supervised learning, the labels are effectively utilized in conjunction with the inherent information present in the data. Jia et al. [47] introduced a semi-supervised Siamese network called 3DAES. This network combines an autoencoder module with a Siamese network to analyze the information contained in a substantial volume of unlabeled data, rectifying it using a small collection of labeled samples. Corrections can effectively mitigate numerous issues arising from limited training data. 

Active learning is a method that involves selecting the most valuable samples from an unlabeled dataset for labeling. These samples are typically the ones about which the model is most uncertain, most representative, or most important to the classification boundary. By labeling these samples, the benefit of each labeling effort is maximized, improving the model’s learning efficiency and classification accuracy [48,49,50]. Active learning algorithms commonly employed in remote sensing include disconnection [51], normalized entropy [52], uncertainty sampling [53], and marginal sampling [54]. In HSI classification, a significant number of labeled samples may be redundant and unnecessary. Through active learning, it is possible to reduce annotation costs [55]. Haut [56] designed a Bayesian convolutional neural network (B-CNN); this method performs active learning on the proposed B-CNN based on a three-step training phase, thereby addressing the difficulties caused by high-dimensional data and overfitting. 

In small-sample HSI classification, the limited size of the training sample set often restricts the classification model due to insufficient information, which leads to inaccurate model classification results [57]. Model training errors subsequently influence sample query strategies, which results in the selection of suboptimal candidate samples based on misclassification information. Lei et al. [58] introduced a novel rank learning loss function into an active learning model, forming an uncertainty predictor, and achieved end-to-end uncertainty learning. Wang et al. [59] introduced a dual-branch domain adaptation few-shot learning (DBDAFSL) method to transfer knowledge obtained from a source domain to a completely different target domain efficiently. Wang et al. [60] proposed a collaborative active learning (CAL) scheme that takes into account the uncertainty and diversity of actively selected samples, as well as the cost of expert annotations. 

While the aforementioned models have achieved positive results in the field of HSI classification, the crucial challenge remains in leveraging a limited amount of training sample data to extract deep feature information effectively and enhance classification accuracy. Our goal is to address the issue of decreased prediction accuracy in scenarios with few or extremely few samples. We recognize that models struggle to learn complete features from a limited number of training samples, leading to prediction biases. Therefore, on the one hand, we leverage contrastive learning and pseudo-active learning to fully explore the type features of both labeled and unlabeled samples. On the other hand, we focus on the quality of training samples by employing active learning methods to query representative samples, helping the model learn comprehensive features. We designed a novel multi-strategy triplet network classifier (MSTNC) in this study. This multi-strategy learning method optimizes multiple related tasks simultaneously, leveraging shared information between tasks to improve the model’s generalization ability and classification performance. Inspired by [42], we first designed a triplet network classifier as the backbone, which includes contrastive and classification modules, with the TNC used for model fine-tuning and classification.

Secondly, we consider ways to improve the classification accuracy of the model without increasing the cost of sample labeling from the perspective of the training strategy. To address the limitation that traditional active learning only considers the sample values on one side of the decision boundary, inspired by [61], a feature-mixture-based active learning (FMAL) method is proposed. This approach aims to explore more valuable samples, enabling the model to learn richer and more detailed features. 

Finally, a dual-strategy pseudo-active learning method is proposed to screen out samples with high accuracy and diversity, which can provide more feature information for the model and effectively compensate for the lack of samples in the training set. Regarding the pseudo-active learning method, we provide a detailed introduction in Section 3.4.

Through the joint training of these strategies, the small-sample hyperspectral image classification method can fully utilize the potential of data and models, improve classification accuracy and robustness in situations with extremely limited samples, and overcome the limitations of traditional methods in small-sample environments.

The main contributions of this article are summarized as follows:

(1) Addressing the challenge of HSI classification with small samples and multiple classes, we employ a composite three-input neural network structure to enhance the capability of capturing intra-class and inter-class features. This method expands the triple sample pair to optimize the model’s classification performance. The triplet network composed of the three-input neural network, projection head, and classifier is used as the backbone for feature extraction and classification tasks.
(2) In this paper, a novel active learning method based on feature mixing is proposed. By blending certain features of labeled samples into unlabeled samples, the predicted changes in the mixed samples can reveal new features in the unlabeled samples. With FMAL, the dependence of sample selection on the quality of the initial model is reduced; thus, representative samples with high information richness are selected. Analytical experiments verify that our method is more effective than the classical methods.
(3) We propose a dual-strategy pseudo-active learning method. Two filters were used to select valuable samples in unannotated samples as pseudo-samples. The two filters adopt different filtering strategies, and the aim is to identify samples with both certain confidence and certain new features. Pseudo-samples are added to the training set, enriching it to improve the accuracy of the model without increasing the labeling costs. The results show that compared with several existing state-of-the-art methods, the MSTNC strategy enhances the overall accuracy in the three generic datasets by 8.34–28.22%, 2.34–12.19%, and 7.07–23.66%, respectively, under the condition of limited training samples (five samples per class).

The remainder of this paper is organized as follows. Section 2 introduces the basic concepts of the triplet network and features mixture-based active learning as well as pseudo-active learning methods. A detailed description of the proposed MSTNC model and the sample construction process is provided in Section 3. In Section 4, a series of elaborately designed experiments validate the effectiveness and necessity of the strategies employed in the proposed model. Section 5 provides a discussion about the proposed method. Finally, Section 6 summarizes the conclusions and provides prospects for future research.

## 2. Related Work 

### 2.1. Triplet Network 

Triplet network (TN) models [42] are primarily applied in situations where there are numerous (or uncertain) sample classes; at the same time, the number of samples in the training dataset is limited (this is exactly the problem facing HSI classification). The TN comprises three identical feed forward networks (with shared parameters). The input consists of a triplet composed of three different samples: a reference sample (x), a sample from the same class ($x_{-}$), and a sample from a different class ($x_{+}$). Their relationship is represented by the Euclidean distance. The training parameters bring x closer to $x_{+}$ and away from $x_{-}$, thus enabling the classification task. Triplet loss is expressed as follows:

$$L=max\left(d\left(x,x_{+}\right)-d\left(x,x_{-}\right)+margin,0\right),$$

where *d*(·) represents the Euclidean distance.

This similarity-based approach allows for a more detailed characterization of differences between different classes without increasing the number of labeled samples. Consequently, it facilitates better capture of both intra-class and inter-class features. Florian Schroff et al. [62] used the triplet network for the face recognition system. Chengliang Liu et al. [63] used the triplet network for tactile grasp outcomes prediction.

In this study, we applied the TN to the field of HSI classification and enriched the TN structure by integrating the TN and the classifier into a framework that mainly consists of an encoder, a classifier, and a projection head. The data dimension of hyperspectral images is extremely high and contains a lot of redundant information. The encoder maps high-dimensional data to a lower dimensional latent feature space through the multi-layer structure of deep neural networks. This can not only effectively reduce the dimensionality of the data but also improve the performance of the classifier by learning to capture high-order feature representations in spectral and spatial information. The main purpose of the projection head is to map feature vectors from a high-dimensional feature space to another target space (usually a low-dimensional space). In the process of contrastive learning, the projection head can project the feature vector into a new space to perform more effective feature comparison in the training phase to improve, in turn, the model’s ability to extract potential features. A small number of labeled samples can be used to create a large number of triple training samples, expanding the training set. Projection head contrastive training and classifier classification training are performed alternately so that the model can learn richer feature representations so as to maximize the classification accuracy of the model. 

### 2.2. Active Learning 

Active learning is a method that involves selecting the most valuable samples from an unlabeled dataset for labeling. These samples are typically the ones about which the model is most uncertain, most representative, or most important to the classification boundary. By labeling these samples, the benefit of each labeling effort is maximized, improving the model’s learning efficiency and classification accuracy [64,65,66]. Ajay J et al. [67] proposed an uncertainty measure that generalizes margin-based uncertainty to the multi-class case and is easy to compute. Because this approach relies on both the best guess and the second-best guess, it is referred to as the best and suboptimal (BvSB) method. If the probability difference between the best and second-best categories for a sample is small, it means that the classifier is highly uncertain about this sample, indicating that it has a higher value for labeling. This is a commonly used classic active learning method. 

Cao [68] integrated the best-versus-second-best (BvSB) method with a CNN into a framework, leveraging the CNN’s powerful feature extraction ability and the annotation efficiency of active learning. 

Despite the effectiveness of the above method, it is still difficult when applied to deep neural networks, high-dimensional data, and low-data states. It remains to be explored whether active learning methods based on posterior probability can identify truly valuable samples under the premise of minimum labeling cost.

Amin Parvaneh et al. [61] introduced an active learning method called ALFA-Mix, which constructs interpolations between the representations of labelled and unlabeled samples and then examines the predicted labels. This method identifies unlabeled samples with sufficiently distinct features by seeking inconsistencies in predictions resulting from interventions on their representations. 

Specifically, the following conclusions and methods are presented:

1. The characteristics of the latent space play a crucial role in identifying the most valuable samples to be labeled.
2. The model’s incorrect predictions mainly stem from novel “features” in the input that are not recognizable.
3. Interpolation between the representations of unlabeled and labeled instances is adopted to achieve sampling of new instances, without explicitly modeling the joint probability of labeled and unlabeled instances [69,70,71,72].
4. The model predicts the loss of the pseudo-label of an unlabeled instance at its interpolation with a labelled one. By utilizing losses, it is possible to calculate which features are novel to the model.

In small-sample HSI classification, the limited size of the training sample set often restricts the classification model due to insufficient information, which leads to inaccurate model classification results [73]. Model training errors subsequently influence sample query strategies, resulting in the selection of suboptimal candidate samples based on misclassification information. To address the challenges of small-sample HSI classification, this paper draws on the theories and methods and introduces a feature-mixing-based active learning (FMAL) method. We refer to the feature vectors output by the encoding layer as encoding-layer features. Encoding-layer features play a crucial role in identifying valuable unlabeled samples. The changes in the prediction values of the model are observed by mixing the encoding-layer features of the labeled samples with the encoding-layer features of the unlabeled samples. These changes in predicted values can assist the model in discovering new features within unlabeled samples that are overlooked or unrecognized by the model. Subsequently, valuable samples can be selected based on these discoveries. The samples queried by the FMAL method contain substantial information and strong representation, effectively improving the model’s accuracy while working with a small-sample dataset.

### 2.3. Pseudo-Active Learning

The pseudo-label model [74], as a simple and effective semi-supervised learning method, has two core ideas:

1. Using the trained model to give unlabeled data a pseudo label. The method is very straightforward: use the training model to predict unlabeled data and use the category with the highest probability as the pseudo label for unlabeled data.
2. Applying entropy regularization to transform unsupervised data into regularization terms of the objective function (loss).

Haofeng Zhang et al. [75] used pseudo-labelling for image retrieval. Wenying Zhu et al. [76] used pseudo-labelling for bearing fault diagnosis.

HSIs contain a significant number of unannotated pixels that can be used as candidate pseudo-label samples. In this study, we combine the above ideas into model training and propose a pseudo-active learning strategy.

Distinguishing from AL with oracle labeling, we label unlabeled samples based on the model’s predicted values. The predicted values of unlabeled samples generated by the classifier are referred to as pseudo-labels. The samples labeled with the predicted values of the classifier are called pseudo-labeled samples or pseudo-samples.

Using pseudo-label samples may decrease the model’s performance. This is mainly due to the following two reasons:

1. Noise caused by incorrect labeling of pseudo-label samples;
2. The lack of new features between the pseudo-label samples and the training set samples, leading to model overfitting.

To reduce noise interference, it is necessary to select samples with high confidence. Meanwhile, in order to avoid overfitting, it is necessary to select samples with high uncertainty. In order to meet the needs of both aspects, a new pseudo-label filtering method, the dual strategy dual threshold filtering method, is proposed.

After screening, the pseudo-labeled samples that meet the criteria are merged into the training set for retraining. This approach effectively expands the training set and improves model classification accuracy without increasing the cost of sample labeling.

## 3. Materials and Methods

### 3.1. The MSTNC Framework

**Figure 1:**
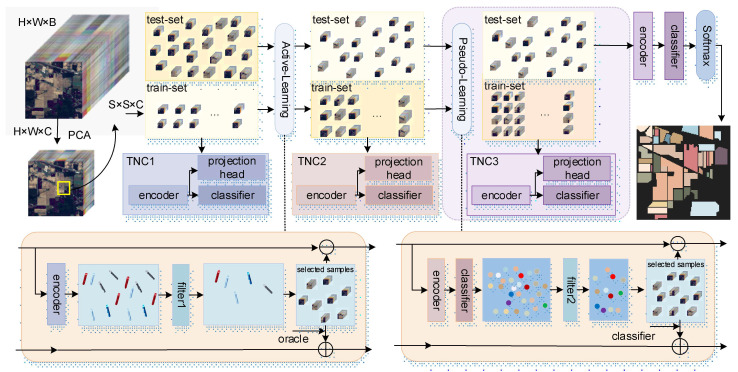
The overall architecture of the proposed MSTN for HIS classification. 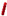: encoding layer vector of the sample; 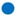: the classifier output vector of the sample; ⊕: add selected sample to the train-set; ⊖: remove selected sample from the test-set; filter 1: a filter used in active learning strategies to screen samples using the FMAX method; filter 2: a filter used in the pseudo-active learning strategy, which includes two filters that filter high-confidence samples and high-uncertainty samples, respectively.

This study proposes a multi-strategy learning method based on triplet networks. As shown in Figure 1, given an HSI dataset denoted as $H\;\epsilon \;R^{H\times W\times B}$, where H, W, and B represent the height, width, and spectral dimensions of the image, respectively, we first applied traditional principal component analysis (PCA) [77] to the spectral bands of the initial HSI data to reduce the number of spectral bands, resulting in dimensionality-reduced HSI data with spectral dimension *C*, denoted as $I\;\epsilon \;R^{H\times W\times C}$. This process retains the critical spectral information for classification while reducing the computational load. The height and width of the original data remain unchanged, such that it preserves the spatial information, which is crucial for recognizing any object. The HSI data cube (*I*) is divided into small overlapping 3D patches $O\;\epsilon \;R^{S\times S\times C}$, where *S* denotes the height and width, and the truth-label of each small patch is determined by the label of the center pixel. These small 3D patches cover the *S* × *S* window spatial extent and all *C* spectral bands and constitute the original training and test sets. The TNC serves as the backbone and is trained with samples from the original training set to obtain TNC1. Subsequently, FMAL utilizes the encoding-layer feature vectors generated by the encoder part of TNC1 to query the most valuable unlabeled samples in the test set for oracle labeling. The labeled samples are removed from the test set and added to the training set, and the TNC is retrained to obtain TNC2. TNC2 is used to predict the remaining samples in the test set, and the predicted values serve as labels for the sample. The high-quality samples that meet the requirements are selected by the PAL strategy and added to the training set. Then, the latest training set is used to train the TNC, resulting in TNC3. Finally, the softmax calculation is performed on the classifier results to output the classification results.

### 3.2. Triplet Network Classifier

**Figure 2:**
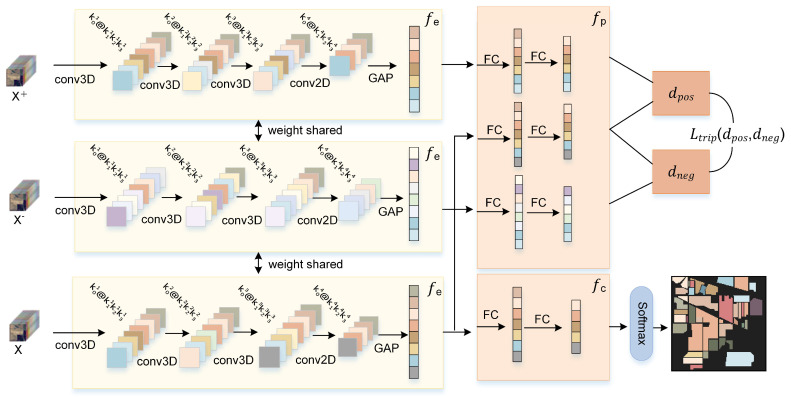
TNC detailed model structure. $k_{0}^{i}$@$k_{1}^{i}k_{2}^{i}k_{3}^{i}$: $k_{0}^{i}$ denote the size of the convolution kernel; $k_{1}^{i}k_{2}^{i}k_{3}^{i}$ represents the three dimensions of the input data; i represents the *i*-th layer convolution.

As shown in Figure 2, the TNC in this study comprises three main components: an encoder $f_{e}$, projection head $f_{p}$, and classifier $f_{c}$. The encoder $f_{e}$ consists of three cascaded 3D convolutional layers, followed by a 2D convolutional layer, and finally linked to a global average pooling layer (GAP). Both $f_{p}$ and $f_{c}$ consist of two fully connected (FC) layers.

The contrastive learning module of the TNC network is formed by $f_{p}$ and $f_{e}$. The classification learning module is formed by $f_{c}$ and $f_{e}$. The $f_{c}$ is connected to a softmax layer to output the classification results.

The TNC will be trained as a backbone in two stages. The first stage is the contrastive learning phase. Specifically, in a dataset containing samples of Cls classes of land cover, initially, each class selects n labeled samples $x^{l}$ to form the initial training dataset $D^{train}={\left\{\left(x_{i}^{l},y_{i}\right)\right\}}_{i=0}^{n\times Cls}$, where $y_{i}$ is the label of the *i*-th sample. Let N be the total number of samples and the remaining unlabeled samples $x^{u}$ constitute the test set $D^{test}={\left\{x_{i}^{u}\right\}}_{i=0}^{N-n\times Cls}$. For a sample $x^{l}$, it forms a triplet sample with other samples $x^{l+}$ and $x^{l-}$ in the contrastive training set, where $x^{l+}$ shares the same class as $x^{l}$ (positive pair), and $x^{l-}$ belongs to a different class (negative pair). In this way, the total n×Cls samples of $D^{train}$ can form n×(n−1)×n×(Cls−1) triple samples. These triplet samples constitute a contrastive training set, which greatly enriches the number of training samples. To ensure training efficiency, not all samples in the contrastive training set will be used for contrastive learning; instead, a randomly selected number of samples, as a multiple of n×Cls, will be utilized. In the first stage, $f_{e}$ is used to process $x^{l}$, $x^{l+}$, and $x^{l-}$. After processing by $f_{e}$, $z=f_{e}(x)$, three *D*-dimensional encoding-layer feature vectors, z, $z^{+}$, and $z^{-}$, are generated. Encoding-layer feature vectors are crucial for improving model accuracy. These feature vectors are then input into $f_{p}$ to generate three *P*-dimensional feature vectors: v, $v^{+}$, and $v^{-}$. The model is fine-tuned and updated through the triplet loss function, which is described as

(1) $$L_{trip}=max\left(d_{pos}^{2}-d_{neg}^{2}+margin,0\right),$$

(2) $$d_{pos}^{2}={\left\|f_{p}\left(z\right)-f_{p}\left(z^{+}\right)\right\|}_{2}^{2},$$

(3) $$d_{neg}^{2}={\left\|f_{p}\left(z\right)-f_{p}\left(z^{-}\right)\right\|}_{2}^{2}.$$

The ultimate optimization goal is to minimize the distance between positive pairs and maximize the distance between negative pairs. Let $\theta_{e}$, $\theta_{c}$, and $\theta_{f}$ represent the parameters of the encoder $f_{e}$, classifier *f*_*c*_, and projection head *f*_*p*_ in the TNC, which can be updated using Equation (4). During the contrastive learning phase, the parameters $\theta_{c}$ of the classifier $f_{c}$ are not updated.

(4) $$\theta_{e,p}=\underset{\theta_{e,p}}{\mathrm{argmin}}\left\{L_{trip}\left[\begin{array}{c} f_{p}\left(f_{e}\left(x^{l}\right)\right),f_{p}\left(f_{e}\left(x^{l+}\right)\right),\\ f_{p}\left(f_{e}\left(x^{l-}\right)\right);\theta_{e,p} \end{array}\right]\right\}$$

The second stage is the classification learning phase. As illustrated in Figure 2, the input x is processed by the updated $f_{e}$ to generate a *D*-dimensional feature representation vector z. Subsequently, it undergoes processing by $f_{c}$ to produce a Cls-dimensional output vector. Training is guided by computing the cross-entropy loss:

(5) $$L_{class}=-\sum_{i=1}^{Cls}y_{i}*log{\hat{y}}_{i},$$

(6) $$\theta_{e,c}=\underset{\theta_{e,c}}{\mathrm{argmax}}\left\{L_{class}\left[f_{c}\left(f_{e}\left(x_{i}\right)\right),y_{i};\theta_{e,c}\right]\right\},$$

where $y_{i}$ represents the value of the *i*-th class in the ground truth labels (0 or 1) and ${\hat{y}}_{i}$ denotes the probability predicted by the model for the *i*-th class. The parameters $\theta_{e,c}$ of the encoder $f_{e}$ and classifier $f_{c}$ can be updated using Equation (6). During the classification learning phase, the parameters $\theta_{p}$ of the projection head $f_{p}$ are not updated.

After fine-tuning and updating the parameters of the backbone, the final classification results are obtained through the softmax classifier. Joint training of the TNC through the two stages enables the encoding-layer feature vectors to be more conducive to classifying different classes. Simultaneously, this joint learning strategy can enable the model to achieve better classification performance.

### 3.3. Feature-Mixture-Based Active Learning

Active learning can enhance the classification ability of a model by acquiring the most valuable samples with the minimum annotation cost. In the small-sample scenario, the initial performance of the general classifier is poor. The active learning method based on posterior probability cannot guarantee that the samples queried by it have the strongest uncertainty, and the effect of adding them to the training set to improve the accuracy of the model is not ideal. Therefore, we actively select labeled samples to add to $D^{train}$ in a gradually released fashion, rather than extracting them all at once. As the accuracy of the model predictions continues to improve, the quality of the queried samples will also gradually improve. 

Specifically, given the total number of samples M that can be chosen. S is the set of the number of target samples to be identified in each iteration. $S=\left\{s_{0},s_{1},s_{2},\cdots s_{i}\cdots \right\}$, where $s_{i}=min\{M-\sum_{k=0}^{i-1}s_{k},\sum_{k=0}^{i-1}s_{k}\}$, $s_{0}$ represents the number of samples in the initial training set, and $s_{i}$ denotes the number of samples selected in the i-th iteration. The iteration concludes when the number of samples in the training set reaches M. $s_{0}$ samples are initially randomly selected as the training set (and the rest as the test set) to train the model TNC. Then, the active learning method is used to select $s_{1}$ samples in the test set; these samples are removed from the test set and then labeled and added to the training set. Subsequently, the model is retrained using the updated training set. Then, the active learning method is used to select $s_{2}$ samples in the test set, and these samples are removed from the test set, labeled, and added to the training set. The model is then retrained using the updated training set. The above process is repeated until the number of training set samples reaches M. In this way, the M most valuable samples can be selected. The accuracy of the model trained with the training set composed of these samples is higher than that of the training set composed of randomly selected samples.

**Figure 3:**
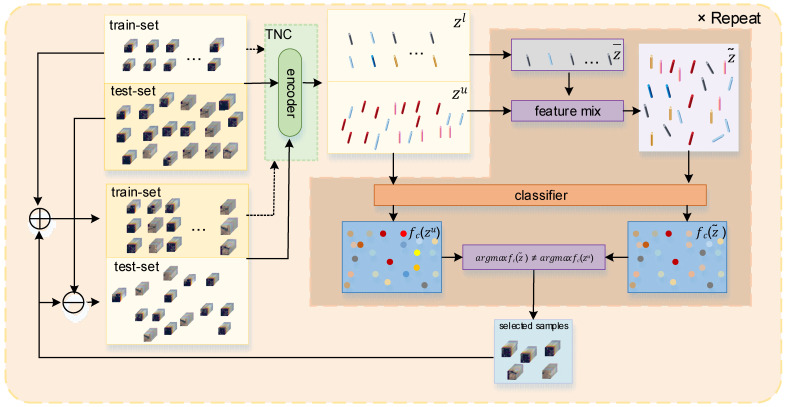
Feature mixing-based active learning (FMAL) illustration. ⊕: add selected sample to the train-set; ⊖: remove selected sample from the test-set; Repeat: number of iterations; 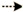: training TNC with train-set.

The iteration process is illustrated in Figure 3.

In the following, the sample selection method in each iteration is elaborated in detail. We refer to the feature vectors output by the encoding layer as encoding-layer features. Encoding-layer features play a crucial role in identifying valuable unlabeled samples. This is because the encoding layer, typically positioned in the middle of deep learning models, extracts high-level features from the input data. Compared to raw data or primary features, these high-level features can better capture the essence and complex patterns of the data. Therefore, encoding-layer feature vectors can provide richer and more meaningful representations than the original spectral data. Secondly, raw hyperspectral data typically have very high dimensions and may contain a lot of redundant information. The encoding layer, through the training process of the neural network, can effectively reduce the dimensionality, remove redundant information, and retain only the most important features that represent class differences. These main features after dimensionality reduction are more concise and clearer, highlighting the essential differences between different classes. Last, but not least, encoding-layer feature vectors can be used to calculate the similarity and differences between samples, as well as the model’s uncertainty in predicting a particular sample. 

We draw on [50] for the feature mixing method by mixing the encoding-layer features of labeled samples with those of unlabeled samples to observe changes in the prediction values of the unlabeled samples. 

If the model’s predictions remain unchanged, it indicates that the proportion of uncertain features in the sample is relatively low, which suggests that there are fewer novel features in the sample. Conversely, if the predictions change, it suggests that the sample contains a sufficient number of novel features, which meet the criteria for the samples we are seeking.

Specifically, we calculate the average value of encoding-layer features of each class of labeled samples in $D^{train}$ as the representative value for each class of labeled samples, which is referred to as the anchor value and denoted as $\bar{z}$. $z^{u}$ represents the encoding-layer feature representation of the samples in $D^{test}$.

We mix each $\bar{z}$ of different classes with each $z^{u}$ in a certain proportion:

(7) $${\tilde{z}}_{i}=\alpha_{i}{\bar{z}}_{i}+\left(1-\alpha_{i}\right)z^{u},\;\alpha_{i}\in {\left[0,1\right.)}^{D},\;{\left\|\alpha_{i}\right\|}_{2}\leq \varepsilon ,\;\;argmaxf_{c}\left(z^{u}\right)=k,i=\left(1,2,\ldots k-1,k+1,\ldots cls\right),$$

where ${\bar{z}}_{i}$ represents the anchor value of the *i*-th class of samples and $\alpha_{i}$ acts as a weight determining the proportion of features that are chosen to be mixed. $\alpha_{i}$ is specific to ${\bar{z}}_{i}$ and $z^{u}$.

For each sample $x^{u}$ in $D^{test}$, we compute its encoding-layer feature $z^{u}$ (assuming its model prediction is k, i.e., $argmaxf_{c}\left(z^{u}\right)=k$). Then, we mix $z^{u}$ with all anchor values except for class k using certain proportions ($\alpha_{i}$). Let ${\tilde{z}}_{i}$ denote the mixed feature value. If the model predictions for all ${\tilde{z}}_{i}$ remain unchanged and still belong to class k, this indicates high consistency between this sample and the k-class samples in the training set. However, if the prediction of any ${\tilde{z}}_{i}$ changes, i.e., ∃i ($argmaxf_{c}\left({\tilde{z}}_{i}\right)\neq k$), this suggests that this sample may contain a sufficient number of new features. This is the sample with training value that we need to retrieve.

ε is the threshold controlling the mixing proportion, and it determines the uncertainty of the selected sample. If ε is smaller, then the selected samples have stronger uncertainty, which means a greater degree of novel features in the samples.

The selection of $\alpha_{i}$ is specific to the input and determines the features to be chosen. Given the *ε* threshold, $\alpha_{i}$ should be chosen to induce a change in the prediction of the model for $\tilde{z}$. We study the impact of feature mixing on the prediction of $z^{u}$ by examining the changes in the loss function.

Using a first-order Taylor expansion with respect to $z^{u}$, the loss of the model for predicting the class of ${\tilde{z}}_{i}$ as k can be rewritten as

(8) $$L_{cls}\left(f_{c}\left({\tilde{z}}_{i}\right),\;y_{k}\right)\approx L_{cls}\left(f_{c}\left(z^{u}\right),\;y_{k}\right)+{\left({\tilde{z}}_{i}-z^{u}\right)}^{T}\cdot {grad}_{z^{u}}L_{cls}\left(f_{c}\left(z^{u}\right),y_{k}\right).$$

From Equations (7) and (8), we obtain

(9) $$L_{cls}\left(f_{c}\left({\tilde{z}}_{i}\right),y_{k}\right)-L_{cls}\left(f_{c}\left(z^{u}\right),y_{k}\right)\approx (\alpha_{i}{\left({\bar{z}}_{i}-z^{u})\right)}^{T}\cdot {grad}_{z^{u}}L_{cls}\left(f_{c}\left(z^{u}\right),\;y_{k}\right).$$

The choice of $\alpha_{i}$ determines the features to be selected with the aim of maximizing the change in loss:

(10) $$\alpha_{i}^{*}=\underset{{\left\|\alpha_{i}\right\|}_{2}\leq \varepsilon }{\mathrm{argmax}}{\left(\alpha_{i}({\bar{z}}_{i}-z^{u})\right)}^{T}\cdot {grad}_{z^{u}}L_{cls}\left(f_{c}\left(z^{u}\right),y_{k}\right).$$

The optimal value $\alpha_{i}^{*}$ is determined by satisfying the threshold constraint, which maximizes the change in the loss function. $\alpha_{i}^{*}$ is calculated as follows:

$${\left\|\alpha_{i}({\bar{z}}_{i}-z^{u})\right\|}_{2}\leq {\left\|\alpha_{i}\right\|}_{2}\cdot {\left\|{\bar{z}}_{i}-z^{u}\right\|}_{2}.$$

Substitute the constraint ${\left\|\alpha_{i}\right\|}_{2}\leq \varepsilon$.

$${\left\|\alpha_{i}({\bar{z}}_{i}-z^{u})\right\|}_{2}\leq \varepsilon {\cdot \left\|{\bar{z}}_{i}-z^{u}\right\|}_{2}\;\mathrm{Let}\;\alpha_{i}^{\prime }=\alpha_{i}({\bar{z}}_{i}-z^{u}),\;\varepsilon^{\prime }=\varepsilon {\cdot \left\|{\bar{z}}_{i}-z^{u}\right\|}_{2},\;\mathrm{and}\;\;{grad=grad}_{z^{u}}L_{cls}\left(f_{c}\left(z^{u}\right),\;y_{k}\right).$$

Equation (10) can be rewritten as

$$({\alpha_{i}^{\prime })}^{*}=\underset{{\left\|\alpha_{i}^{\prime }\right\|}_{2}\leq \varepsilon^{\prime }}{\mathrm{argmax}}{\left(\alpha_{i}^{\prime }\right)}^{T}\cdot grad.$$

This is solved using the dual norm formula:

$$({\alpha_{i}^{\prime })}^{*}=\varepsilon^{\prime }\cdot \;\frac{grad}{{\left\|grad\right\|}_{2}}.$$

Substitute $\alpha_{i}^{\prime }$, $\varepsilon^{\prime }$:

(11) $$\alpha_{i}^{*}=\frac{\varepsilon \cdot {\left\|{\bar{z}}_{i}-z^{u}\right\|}_{2}}{{\left\|grad\right\|}_{2}}\cdot \frac{grad}{\left({\bar{z}}_{i}-z^{u}\right)}.$$

The size of ε determines the quantity of samples identified, and the required number of samples is found step by step by means of fine-tuning ε:=ε+∆ε. Our experiments demonstrate that using this method effectively controls the number of samples identified while maintaining low computational complexity.

### 3.4. Dual-Strategy Pseudo-Active Learning

After the training with the FMAL strategy, the model acquires a certain level of classification ability. At this point, a significant portion of the model’s predicted values for the samples in the test set are accurate, which means that the number of correctly predicted samples is much larger than the number of samples in the training set. Moreover, the correctly predicted samples often include new features distinct from those found in the training set. 

The above analysis inspires us to consider the possibility of using a strategy to identify a certain proportion of high confidence samples and then using a strategy to identify semantically rich samples within these samples. The predicted values of the model are used as labels to label these samples, and then these samples are expanded to the training set. We refer to this novel method as dual-strategy pseudo-active learning (DSPAL). Unlike AL with oracle labeling, we label unlabeled samples using the model’s predicted values. 

The goal of DSPAL is to expand the training set using pseudo-labeled samples and then retrain the model to further improve the model’s accuracy. Obviously, the quality of pseudo-label samples has a significant impact on the performance of the model, so it is necessary to design a high-quality pseudo-label sample selection strategy. 

**Figure 4:**
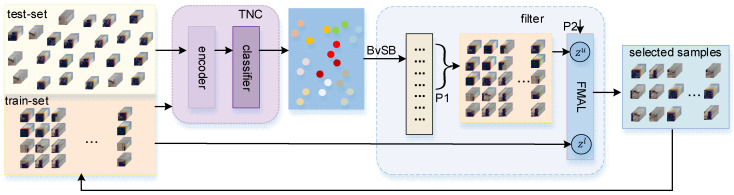
The proposed DSPAL architecture. A filter is established to screen samples classified by the classifier. The samples are labeled using the predicted values of the classifier, and the qualified pseudo-labeled samples are then added to $D^{train}$ for iterative training.

To enable the model to learn reliable representations of the data during the DSPAL phase, an iterative training approach is adopted. At each iteration, the filter adds filter-qualified pseudo-labeled samples to $D^{train}$ and then retrains the model with an updated $D^{train}$. The DSPAL training process is illustrated in Figure 4.

The filter sets two thresholds, $P_{1}$ and $P_{2}$, which represent the $P_{1}\%$ of high-confidence samples and $P_{2}\%$ of valuable samples selected for each class of unlabeled samples, respectively.

When selecting high confidence samples, we choose to use the BvSB [65] measure. The BvSB measure is specifically designed for multi-class classification problems. Its core idea is to determine the confidence level of the predicted value by comparing the difference in prediction probabilities between the best and second-best classes of the model. The greater the difference, the higher the confidence level; conversely, the smaller the difference, the lower the confidence level. The BvSB values for all samples in $D^{test}$ are computed.

(12) $$BvSB=P_{B}\left(x^{u}\right)-P_{SB}\left(x^{u}\right)$$

Here, $P_{B}\left(x^{u}\right)$ and $P_{SB}\left(x^{u}\right)$ represent the conditional probabilities of sample $x^{u}$ belonging to the optimal and suboptimal classes, respectively.

A greater BvSB value indicates greater confidence in the $f_{c}$ classification results, which suggests more accurate predictions. Conversely, a smaller BvSB value signifies greater sample uncertainty, making it more likely to contain new features.

(13) $$Q_{i}=sort\left({BvSB}_{i}\left(x^{u}\right)\right)$$

${BvSB}_{i}$ denotes the BvSB value of the *i*-th class sample, and $Q_{i}$ denotes the queue with the BvSB values of the *i*-th class samples in descending order. Among them, the samples whose position is less than $P_{1}\%$ in the queue are regarded as reliable samples.

We chose the FMAL’s feature mixing method as the filter’s strategy for selecting samples with new characteristics. Among the high-confidence samples selected above, the FMAL screening method is used to select the $P_{2}\%$ of samples with novel features in each class. It should be noted that the anchor values used here are calculated from the updated training set. 

The filter uses these two strategies to filter out samples from $D^{test}$ that meet these criteria, yielding candidate samples with high reliability and strong uncertainty. These samples are then added to $D^{train}$ and used to train the TNC with the updated training set. 

The above process is repeated to improve the model accuracy by continuously expanding the number of samples in the training set.

The complete procedures of our MSTNC are summarized in Algorithm 1.
**Algorithm 1.** MSTNC model.**Input: HSI patch x ∈** $R^{S\times S\times C}$**, The total number of samples is denoted as M.** **Initialization: train-set:** $D^{train}$**, test-set: **$D^{test}$**1. Obtain**: Triplet samples ($x_{i}^{l},x_{i}^{l+}$, $x_{i}^{l-}$) **2.** Train the TNC with $D^{train}$, Obtain the model TNC1**3. while**: $D^{train}$ Number of samples < M: **4.**    Compute the anchor values $\bar{z}$ for $D^{train}$ **5.**    Encoding-layer features mixing ${\tilde{z}}_{i}=\alpha_{i}{\bar{z}}_{i}+(1-\alpha_{i})z^{u}$**6.**  Put all $x^{u}$ corresponding to $z^{u}$ that meet the condition $argmaxf_{c}(\tilde{z})\neq argmaxf_{c}(z^{u})$ into $D^{train}$, update $D^{train}$, $D^{test}$
**7.**    Train TNC with the updated $D^{train}$ to obtain the model TNC2 **8. while**: iterations < 4 **9.**    Calculate the BvSB values f $D^{test}$ **10.**  Sorting the BvSB values for each class of samples. $Q_{i}=sort({{BvSB}_{i}(x}^{u}))$ **11.**  Put the samples in Q between 0 and $P_{1}$ into candidate set $D^{cand}$ **12.**  **while**: $D^{train}$ Number of samples < $P_{2}$: **13.**       Compute the anchor values $\bar{z}$ for $D^{train}$ **14.**       Encoding-layer features mixing${\tilde{z}}_{i}=\alpha_{i}{\bar{z}}_{i}+(1-\alpha_{i})z^{cand}$**15.**          Put all $x^{cand}$ corresponding to $z^{cand}$ that meet the condition $argmaxf_{c}\left(\tilde{z}\right)\neq argmaxf_{c}\left(z^{u}\right)$ into $D^{train}$, update $D^{train}$
**16.**     Train TNC with the updated $D^{train}$ and obtain the model TNC3**17. Output**: Predict test sample with the model TNC3

## 4. Results 

### 4.1. Hyperspectral Datasets

**Figure 5:**
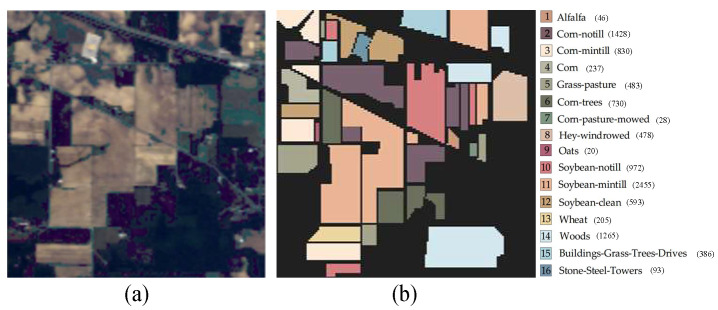
Indian Pines dataset: (**a**) false-color map; (**b**) ground-truth map. The numbers in parentheses represent the total number of samples in each class.

(1) The Indian Pines (IP) dataset was acquired using an airborne visible and infrared imaging spectrometer (AVI-331 RIS). The image size is 145 × 145 pixels, with a wavelength range of 400–2000 nm, a spatial resolution of 20 m, and 200 spectral bands after excluding noisy bands. The dataset contains a total of 10,249 labeled pixels, which span 16 classes, including crops, wood, and other perennial plants. The false-color image and ground-truth map are illustrated in Figure 5.

(2) The Pavia University (PU) dataset was captured over the city of Pavia, Italy, using an airborne hyperspectral imaging spectrometer from Germany. The image has dimensions of 610 × 340 pixels, covers a wavelength range of 430–860 nm, has a spatial resolution of 1.3 m, and consists of 103 spectral bands.

**Figure 6:**
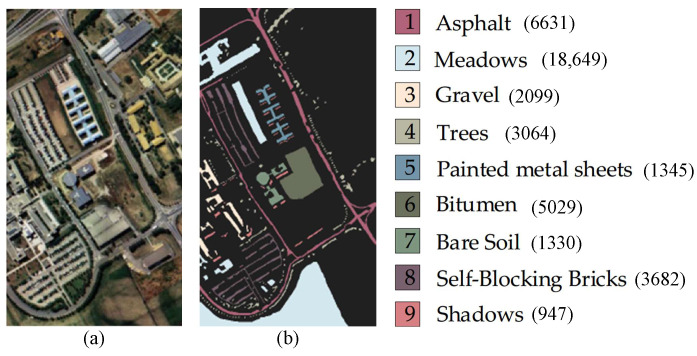
Pavia University dataset: (**a**) false-color map; (**b**) ground-truth map. The numbers in parentheses represent the total number of samples in each class.

The total number of labeled pixels in this dataset is 42,776, and there are nine classes of labeled samples, most of which are urban land cover, such as metal plates, roofs, and concrete pavements. The false-color image and ground-truth map are illustrated in Figure 6.

**Figure 7:**
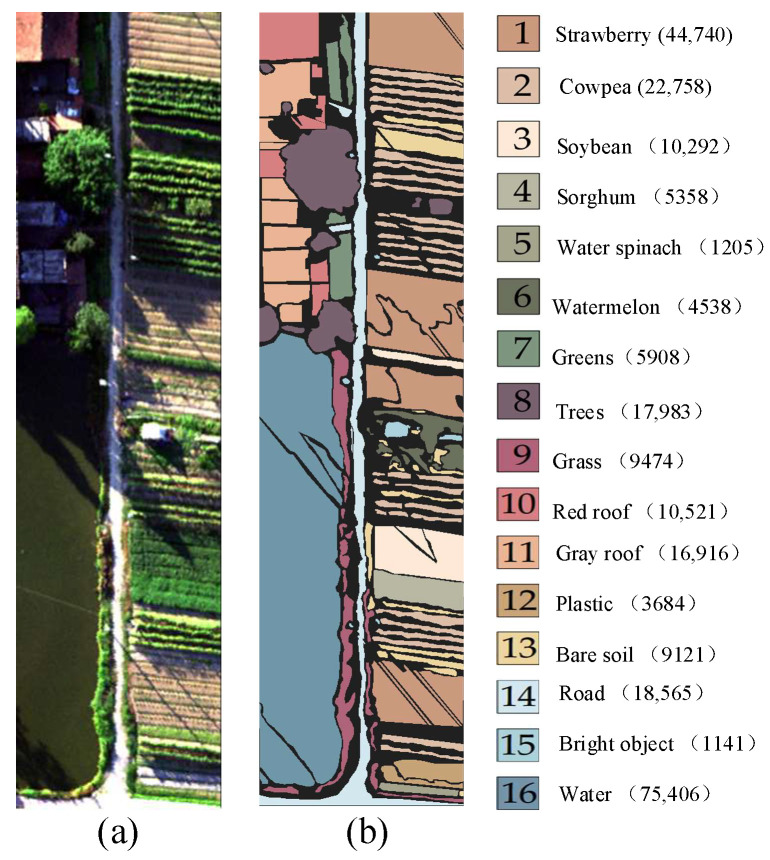
Wuhan Han Chuan dataset: (**a**) false-color map; (**b**) ground-truth map. The numbers in parentheses represent the total number of samples in each class.

(3) The WHU-Hi-HanChuan (WHU) dataset covers the agricultural area of Hanchuan City, Hubei Province, China. The images was acquired by a Headwall nano-hyperspectral imaging sensor mounted on a UAV platform. The scene size is 1217 × 303 pixels with a spatial resolution of 0.109 m. The images were acquired by the Headwall Nano Hyperspectral Imaging Sensor (HNHIS). It consists of 274 bands with wavelengths ranging from 0.4 to 1.0 μm. The ground truth data consisted of 253,580 labeled pixels from 16 different crop type classes. The distribution of the false-color image and ground-truth map are illustrated in Figure 7.

### 4.2. Experimental Settings

All experiments in this paper are conducted on a server equipped with an Intel(R) Core (TM) i7-12700 CPU @ 1.7 GHz 2.19 GHz, 64 GB DDR5 RAM, and an RTX 4090-24 GB GPU. The specific programs were implemented on the Ubuntu operating system using PyTorch 2.0.0 deep learning framework and Python 3.8 compiler. Evaluation metrics include producer accuracy (PA), overall accuracy (OA), average accuracy (AA), and the Kappa statistic (Kappa). Higher values of these metrics indicate that the model is more capable of classifying. Moreover, train time (s) and test time (s) were used for complexity comparison. During the experiments, a batch size of 64 is set, the initial learning rate is set to 0.0001, and the Adam optimizer is employed to facilitate rapid convergence of the model. 

### 4.3. Comparison of Classification Results

To verify the effectiveness of the MSTNC, we studied methods outlined in the literature and designed the following sets of comparative experiments. The choice of comparison methods takes into account its properties and the setup of the original experimental sample.

To ensure a fair comparison, we reproduced all of these methods, and all hyperparameters were also based on publicly available original code or papers. All experiments were conducted using the same hardware, and the ratio of training to test samples was the same. Each datum represents the average of 10 repetitions of the experiment. Table 1, Table 2 and Table 3 give per-class quantitative results for the three datasets.

- Performance with very few training samples

To evaluate the superiority of the proposed method in a few labeled sample scenarios, we selected five samples for each class in the dataset to form the training set. In this way, the proportion of samples in the training set in the three datasets was 0.78% (IP), 0.1% (PU), and 0.03% (WHU). The methods 3D-CNN [20], DFSL-NN [36], DFSL-SVM [36], graph information aggregation cross-domain few-shot learning (Gia-CFSL) [38], dual-branch domain adaptation few-shot learning (DBDAFSL) [59], and CapsGLOM [60] were adopted for comparison with our proposed MSTNC. These HSI classification methods are representative state-of-the-art deep learning methods specifically designed for small sample classification.

The first five methods randomly select five samples per class for experimentation. CapsGLOM uses its original sample selection method; i.e., one sample per class is randomly selected and iterates four times according to the AL method it provided, resulting in the final selection of five samples per class. The MSTNC randomly selects three samples per class and then uses the AL method proposed in this study to select two more samples per class.

Table 1, Table 2 and Table 3 give per-class quantitative results for the three datasets.

From Table 1, Table 2 and Table 3, it can be observed that our proposed MSTNC consistently outperforms other methods in terms of OA, AA, and Kappa on the three datasets. OA is higher by 8.34–28.22%, 2.34–12.19%, and 7.07–23.66%, respectively, AA is higher by 7.31–25.06%, 1.95–16.36%, and 3.58–37.47%, while Kappa is higher by 10.10–28.49%, 3.35–16.64%, and 8.14–29.06%, respectively. Examining the classification accuracy for each class, it can be noted that the IP dataset exhibits its lowest accuracy for class 5 at 69.21%, which surpasses the lowest accuracy of other methods by 24.43–48.98%. For the PU dataset, the lowest accuracy is in class 4 at 71.06%, which surpasses the lowest accuracy of other methods by 16.65–51.6%. In the case of the WHU dataset, the lowest accuracy is in class 12 at 51.94%, which surpasses the lowest accuracy of other methods by 8.51–51.90%.

**Figure 8:**
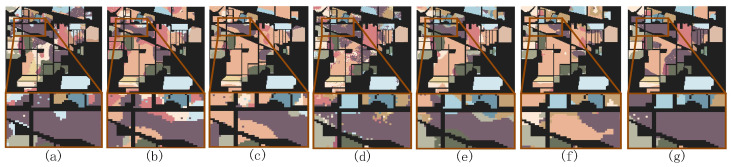
Classification maps of the different models for the Indian Pines dataset: (**a**) 3DCNN; (**b**) DFSL-NN; (**c**) DFSL-SVM; (**d**) Gia-CFSL; (**e**) DBDAFSL; (**f**) CapsGLOM; (**g**) MSTNC.

**Figure 9:**
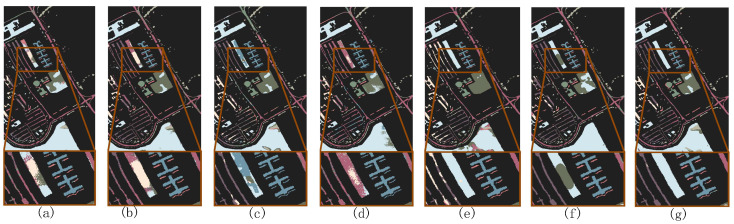
Classification maps of the different models for the Pavia University dataset: (**a**) 3DCNN; (**b**) DFSL-NN; (**c**) DFSL-SVM; (**d**) Gia-CFSL; (**e**) DBDAFSL; (**f**) CapsGLOM; (**g**) MSTNC.

**Figure 10:**
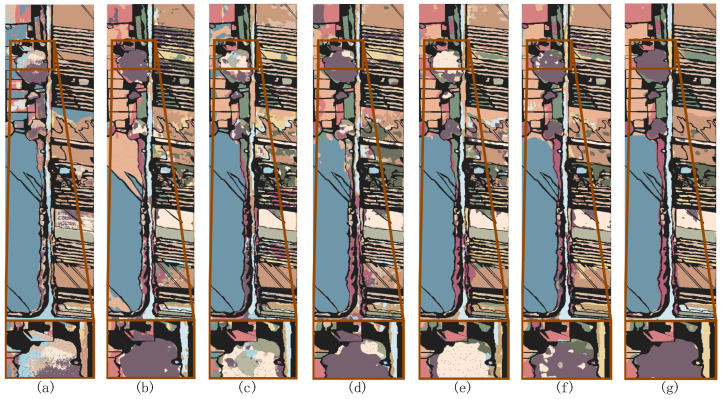
Classification maps of the different models for the WHU-Hi-HanChuan dataset: (**a**) 3DCNN; (**b**) DFSL-NN; (**c**) DFSL-SVM; (**d**) Gia-CFSL; (**e**) DBDAFSL; (**f**) CapsGLOM; (**g**) MSTNC.

Figure 8, Figure 9 and Figure 10 show the classification maps of all the methods on the IP, PU, and WHU datasets. The maps demonstrate that our proposed model exhibits superior classification performance and clearer boundaries. For example, in Figure 8 (IP dataset), it can be observed that other models incorrectly label some pixels of class 2 (Corn—no-till) as class 11 (Soybean—no-till) or class 14 (Soybean—min-till); in Figure 9 (PU dataset), other models exhibit considerable confusion in class 8 (self-blocking bricks); and in Figure 10 (WHU dataset), it is apparent that other models incorrectly classify class 8 (trees) and class 10 (red roof) as other classes. However, the MSTNC achieves considerably high classification accuracy for these classes.

Conclusively, the proposed method shows advantages in improving the classification performance of all datasets. Compared to other methods, the proposed model demonstrates superior classification of background pixels, which affirms the effectiveness of this approach.

B. Performance with limited training samples

To make a broader comparison with the representative and state-of-the-art classification methods under multiple technical routes, we performed the following experiments:

The training set represented 2% (IP), 1% (PU), and 0.5% (WHU) of the categories in each sample, respectively.

The spectral–spatial residual network (SSRN) [31], semi-supervised Siamese network (3DAES) [47], contrastive GCN (ConGcn) [33], TensorSSA [22], and SmaT-HOSVD [23] models were adopted for comparison with our proposed MSTNC.

The sample size selected for this experiment is slightly larger than that of the previous group. Especially for the PU dataset and WHU dataset, as these two datasets are relatively large, the absolute sample size of the training set is also large. Therefore, the accuracy is generally higher than the previous group.

Table 4 provides quantitative results for the three datasets.

As can be observed from Table 4, our proposed MSTNC method has significant advantages over other methods in terms of OA, AA, and Kappa coefficients on all three datasets. On the Indian Pines (IP) dataset, the MSTNC outperforms OA by 3.91–20.2%, AA by 4.4–14.77%, and Kappa coefficient by 2.84–23.36%. For the Pavia University (PU) dataset, the MSTNC achieved 100% for OA, AA, and Kappa, which were 3.12–8.73%, 5.55–11.5%, and 2.12–12% higher, respectively, than the other method. On the WHU dataset, the MSTNC also achieved 100% for OA, AA, and Kappa, improving by 1.99–10.09%, 1.33–8.79%, and 2.05–7.41%, respectively, compared to the other method. By specifically analyzing each dataset, it can be seen that the MSTNC method consistently outperforms other methods in all the metrics, especially in the PU and WHU datasets, where the MSTNC achieves 100% classification accuracy and Kappa coefficient, which further proves the excellent performance of the method in the hyperspectral image classification task.

C. Performance with Different Training Percentages

**Figure 11:**
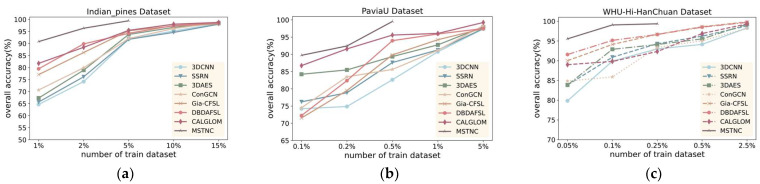
Evolution of OA as a function of the number of training samples per class: (**a**) IP dataset; (**b**) PU dataset; (**c**) WHU dataset.

**Figure 12:**
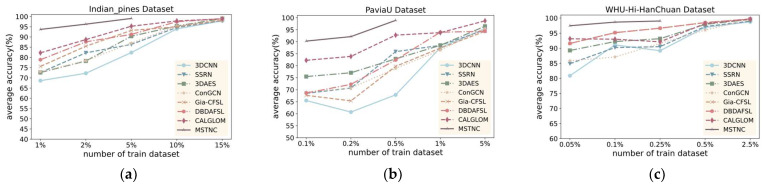
Evolution of AA as a function of the number of training samples per class: (**a**) IP dataset; (**b**) PU dataset; (**c**) WHU dataset.

**Figure 13:**
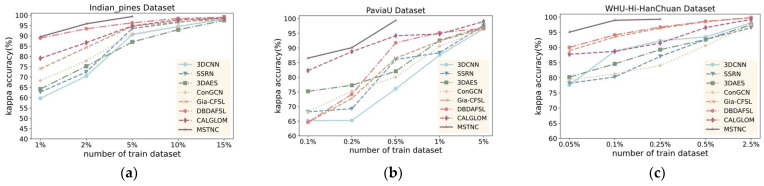
Evolution of Kappa as a function of the number of training samples per class: (**a**) IP dataset; (**b**) PU dataset; (**c**) WHU dataset.

To validate the generalization performance of the proposed MSTNC, we compared the classification results of different methods with different numbers of training samples. Here, we selected the seven most representative deep learning methods in experiments A and B. Considering the great disparity in the number of samples in different datasets, different percentages of training samples were selected. Experiments were conducted with selected samples of 1%, 2%, 5%, 10%, and 15% of the IP dataset; 0.1%, 0.2%, 0.5%, 1%, and 5% of the PU dataset; and 0.05%, 0.1%, 0.25%, 0.5, and 2.5% of the WHU dataset as the training set, with the rest of the samples as the test set. The experimental results are shown in Figure 11, Figure 12 and Figure 13. Our proposed MSTNC consistently achieves the highest accuracy across different sample quantities on the three datasets, which demonstrates its superiority in terms of generalization performance.

From Figure 11, Figure 12 and Figure 13, it can be observed that on the IP dataset, when the proportion of samples is 5%, the MSTNC method achieves more than 99% for all three metrics. It performs 5.11–9.52%, 5.95–16.68%, and 4.70–14.02% better than other methods. Other methods require a sample proportion of 15% to achieve a similar level of accuracy.

On the PU dataset, when the proportion of samples is 0.5%, the MSTNC method achieves over 98% for all three metrics. It performs 3.86–15.68%, 7.01–30.43%, and 5.07–23.21% better than other methods. Other methods require a sample proportion of 5% to achieve a similar level of accuracy.

On the WHU dataset, when the proportion of samples is 0.25%, the MSTNC method achieves over 98% for all three metrics. It performs 3.01–7.86%, 2.28–11.09%, and 3.11–15.15% better than other methods. Other methods require a sample proportion of 2.5% to achieve a similar level of accuracy.

D. Complexity Analysis

In order to control the complexity of the model, we chose a lightweight model structure suitable for small samples to reduce the complexity of the model. GAP is used to replace the fully connected layer to reduce the number of parameters and the amount of computation. At the same time, we adopt the PCA dimensionality reduction technique to further reduce the dimension of the input data to reduce the computational overhead.

To balance model complexity and computational efficiency, we fine-tuned the model structure and hyperparameters through multiple experiments. This ensured that we could simplify the model as much as possible without compromising classification accuracy significantly. Taking the IP dataset as an example, the OA of the model is 82.97%. In the case of, we reduce the inference time of the model to 1.32 s by reducing the number of layers and parameters. Thus, a balance between accuracy and efficiency is achieved.

**Figure 14:**
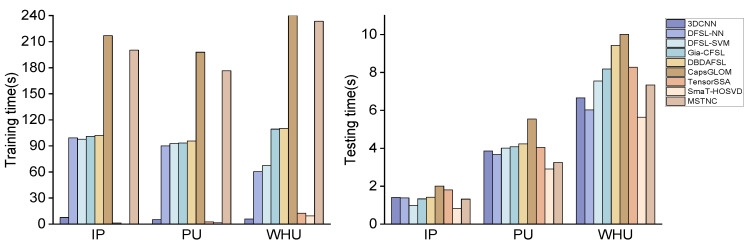
Training time and testing time of different methods.

The training time and test time of different models on different datasets are reported in Figure 14.

From the figure, it is evident that TensorSSA and SmaT-HOSVD boast the shortest training time, significantly shorter than other deep-learning-based methods, thus exhibiting considerable advantages. However, during the testing phase, the disparity narrows considerably, with all methods falling below 12 s. Among the deep learning methods, CapsGLOM and MSTNC necessitate active learning techniques for sample querying, resulting in longer training durations. Nevertheless, their testing times are moderate. Notably, the MSTNC excels in testing efficiency by utilizing learning strategies to enhance model efficiency, coupled with a lightweight classifier.

Unlike ordinary images, a hyperspectral image of a scene often corresponds to a very wide area of space in the actual range. The pixel-level labeling of hyperspectral images involves many complicated works. Therefore, controlling the number of samples and improving the model accuracy is the primary goal of HSI classification. The MSTNC achieves high accuracy in extremely small sample scenarios with moderate time complexity.

### 4.4. Ablation Analysis

In order to eliminate the effect of different training samples on each ablation method, we unified the training samples using fixed random seeds.

#### 4.4.1. Effectiveness of Triplet Contrastive Learning

**Figure 15:**
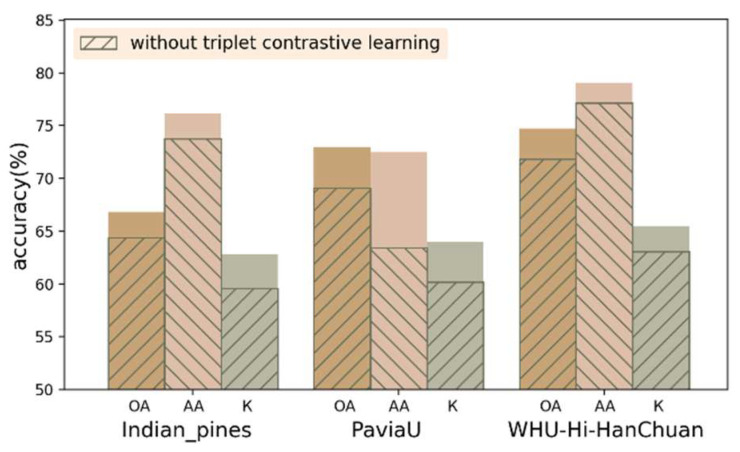
Ablation experiments of the triplet contrastive learning.

To validate the effectiveness of triplet contrastive learning, experiments were conducted to compare the complete TNC with the TNC containing only $f_{e}$ and $f_{c}$. In these experiments, three labeled samples per class were randomly selected for training. The results of the evaluation metrics are shown in Figure 15. On the three datasets, OA improved by 3.24%, 4.21%, and 3.86%, respectively. AA improved by 3.16%, 9.85%, and 2.44%, and Kappa improved by 4.20%, 4.50%, and 2.31%. Thus, the expansion of the triplet training set effectively extracts richer features and information by reducing the intra-class distance and increasing inter-class distance. This approach significantly enhances the model’s classification. The reason why this method is effective is that it uses triple sample pairs in training. A small number of training samples can form enough triple sample pairs, greatly expanding the training set.

#### 4.4.2. Effectiveness of AL and PAL Strategies

To validate the effectiveness of AL and PAL strategies, three sets of experiments were conducted on the IP dataset.

**Figure 16:**
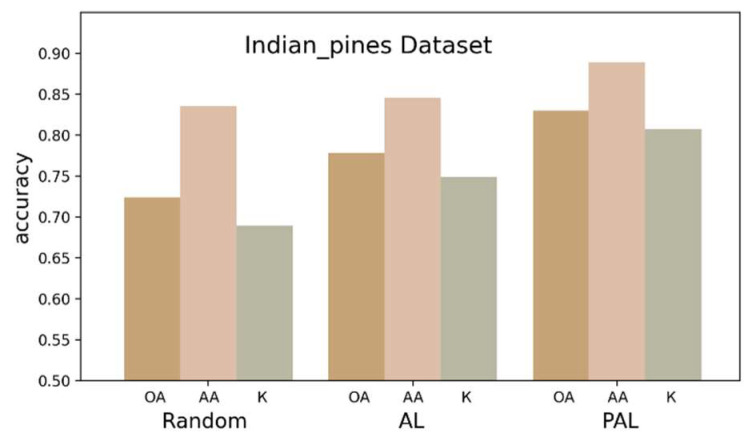
Ablation experiments of the proposed method on.

In the first set, five labeled samples were randomly selected from each class of the IP dataset. In the second set, three samples were first randomly selected from each class, and then two samples were selected by our AL method to form an initial training set for further training. The experimental results in Figure 16 reveal that compared to training with randomly selected samples, the AL method resulted in improvements of 5.59%, 1.10%, and 5.48% in OA, AA, and Kappa, respectively. This proves that the proposed AL strategy is effective, indicating that its selected samples are more efficient. In the third set, the PAL strategy was additionally applied to the training process based on the second set. As shown in Figure 16, compared with AL alone, the OA, AA, and Kappa values increased by 4.26%, 2.95%, and 4.74%, respectively, after PAL application. The results highlight significant improvement in the predictive accuracy with PAL, showcasing its ability to enhance classification accuracy without increasing the cost of labeled samples. This underscores the superiority and feasibility of the proposed method, which confirms that the accuracy and semantic richness of pseudo-labeled samples align with our theoretical expectations. It also demonstrates that the joint application of these two strategies effectively enhances the feature representation of the network, detail capture, and discriminative abilities, particularly in scenarios with extremely limited initial samples.

## 5. Discussion

### 5.1. Parameter Analysis

Deep learning methods can be considered as end-to-end learning methods. End-to-end learning can automatically extract features and perform classification through models such as CNN. This process does not rely on artificial feature selection. This enables the model to adapt to the influence of noise and improves the generalization ability and robustness of the model.

Hyperspectral images are characterized by high dimensionality and severe redundancy. With dimension-reduction processing, important spectral information can be effectively retained, while reducing data redundancy and computational complexity. PCA is one of the most commonly used dimensionality reduction methods. Because it does not rely on sample labels, it not only effectively reduces the dimensionality of spectral bands but also maintains the integrity of spatial information. Therefore, this paper adopted PCA for dimensionality-reduction processing. The high dimensionality of spectral bands is beneficial for information preservation, but it increases redundancy, noise, and the complexity of model training. On the contrary, the dimensionality of spectral bands is low, which may lead to information loss but can help remove redundancy and noise.

After dimensionality reduction, the data are normalized along the spectral dimension to ensure that the mean is 0 and the unit variance is used as the standard. This standardization helps improve the performance and stability of deep learning models. HSI classification algorithms are often trained and classified based on hyperspectral image patches. Hyperspectral image patches are used as input samples and cropped around the central pixel. The selection of patch size primarily considers the extraction of spatial features and computational costs. Increasing the patch size provides a larger perceptual field, which is beneficial for the model. At the same time, it also brings more noise and chaos, which is harmful to the model.

For the MSTNC model, a patch size of 25 × 25 with 30 bands is used for the IP and WHU datasets. On the PU dataset, the patch size is set to 19 × 19, with 15 bands. The chosen patch values and band values are based on reference [47,59,60,78] and extensive experiments [79], and this article continues to build upon the conclusions drawn from previous work.

In the FMAL method, the threshold (ε, ∆ε) affects the execution efficiency of FMAL. ε reflects the quality of the selected samples. The larger the value of ε, the fewer new features the selected sample contains, and vice versa. Therefore, (ε, ∆ε) determines the number of samples identified at each time.

In the DSPAL method, $P_{1}$ and $P_{2}$ are the thresholds of the confidence filter and uncertainty filter, respectively. The larger the $P_{1}$, the lower the confidence of the selected sample and vice versa. The larger the $P_{2}$, the higher the uncertainty of the selected sample, and vice versa.

For the MSTNC model, ε = 0.006, ∆ε = 0.0005, $P_{1}$ = 40, and $P_{2}$ = 39.

The selection of the above four parameters in this article is mainly based on the analysis of experimental data. More scientific methods need to be further explored.

### 5.2. Analysis of Various MSTNC Strategies

We conducted extensive experiments to thoroughly validate the effectiveness of our proposed method.

#### 5.2.1. Comparison of Three Different Sample Selection Methods

**Figure 17:**
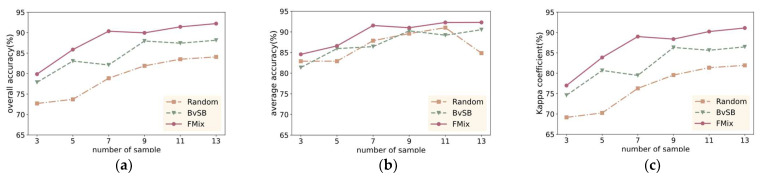
Comparison of accuracy of three different sample selection methods: (**a**) OA; (**b**) AA; (**c**) Kappa.

To validate the superiority of the FMAL method, we compared it with the classical BVSB method and random sample selection method. On the IP dataset, we initially randomly selected 3 samples from each class and then used these 4 methods to choose 3, 5, 7, 9, 11, and 13 samples for training in each class. The experimental results are shown in Figure 17.

The data clearly show that the FMAL method proposed in this paper has the best performance. Specifically, when three labeled samples were selected for each class, the OA, AA, and Kappa of the FMAL method were 2.36%, 3.15%, and 2.47% higher than those of the BvSB method, and 7.09%, 2.28%, and 8.23% higher than those of the random method.

The BvSB method’s OA and Kappa are also superior to the random method, as random sampling may lead to an imbalanced distribution of training samples among different classes, which results in insufficiently representative features for the selected samples. In Figure 17b, the AA values of the BvSB method and the random method repeatedly cross. This may be because the classification accuracy of some classes is low when using the initial model for classification, and the BvSB method heavily relies on the classification results of the initial model, which leads to lower-quality samples for these specific classes. In contrast, FMAL maintains good performance, which indicates that the method of mixing partially labeled sample characteristics reduces the dependence of sample selection on model accuracy.

**Figure 18:**
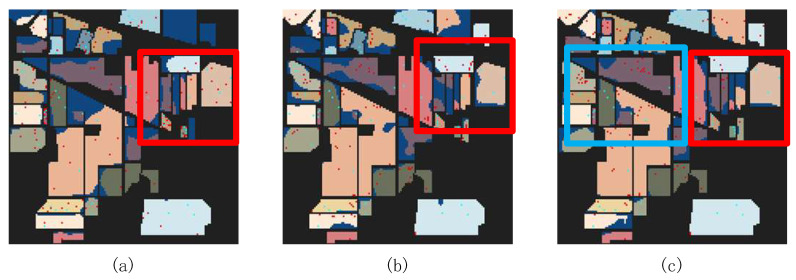
Classification maps of different methods for Indian Pines. Seven labeled samples were selected for each class. Light blue dots are randomly selected samples. The red dots is samples selected with a specific method. Dark blue dots are samples that were incorrectly predicted. (**a**) Random method; (**b**) BvSB method; (**c**) FMAL method.

Figure 18 shows the classification maps when seven samples per class were selected using three different sample selection methods, visualizing the classification effect of the different methods. It is evident that FMAL method exhibits the strongest purposefulness in sample selection, which results in the best classification performance. On the other hand, the random method lacks purpose, leading to it having the poorest performance. Specifically, as can be seen from the red boxed area in Figure 18, the sample selected by FMAL is more representative and the most efficient. Although the samples selected by BVSB show slightly higher relevance in some cases, the efficiency is still better than that of the random method. In addition, the blue boxed area in Figure 18c demonstrates that selecting too many samples at once results in smaller differences between some of the samples, highlighting the importance of iteration.

#### 5.2.2. Analysis of AL Iterative Strategy

To validate the role of iteration in active learning, we conducted experiments using the IP dataset, which is the most representative and has the highest classification difficulty. Initially, 48 samples (3 samples per class) were randomly selected, which served as the initial training set. Then, the number of samples gradually increased using three different methods. AL0 denotes that all desired samples are randomly selected, AL1 denotes that all desired samples are selected using one active learning method, and ALn denotes that the desired samples are selected step by step through iterations.

**Figure 19:**
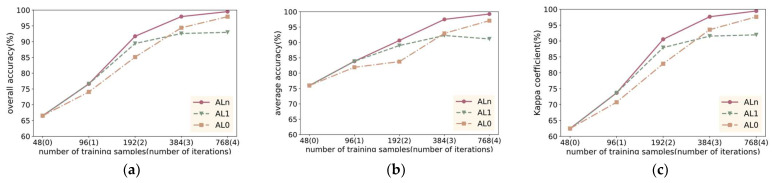
Analysis of the role of iteration in active learning methods. The data in parentheses in the horizontal axis represent the number of iterations of ALn. (**a**) OA; (**b**) AA; (**c**) Kappa.

From the results in Figure 19a–c, it is evident that the accuracy of the model using the Aln method is consistently higher than that of AL0 and AL1. This suggests that model accuracy has an impact on the quality of the samples selected. The iterative approach selects samples on the basis of a model with progressively higher accuracy and therefore achieves better results.

When the number of samples increases to 192, AL1 surpasses the AL0 method, which indicates that FMAL’s purposeful identification of uncertain samples is more effective than randomly selecting samples.

As the number of samples increases to 384, the accuracy improvement rate of AL1 significantly decreases, and during the increment, the accuracy values of AL1 and AL0 cross over, with AL0 subsequently surpassing AL1. The reason behind this phenomenon is that as the number of samples selected by the AL1 method increases, a phenomenon akin to “oversampling” emerges. This leads to high consistency among the chosen samples, which causes the model accuracy to fall even below that of randomly selected samples.

When the total number of samples in the training set reached 768, the classification accuracy of Aln almost reached 100%, maintaining the highest classification accuracy. Simultaneously, examining the change in model classification accuracy in Figure 19 reveals that the accuracy of AL0 approached that of Aln. This is because the coverage of the IP dataset increased significantly when too many samples were selected, leading to smaller and smaller differences in the samples selected. Similarly, the accuracy of AL1 did not significantly decrease for the same reason.

#### 5.2.3. Analysis of the Number of DSPAL Iterations

One iteration of PAL can significantly increase the number of training samples without increasing the sample labeling cost. To analyze the impact of multiple iterations of DSPAL on model accuracy, eight iterations of DSPAL were conducted on models trained through active learning on three datasets. The filter thresholds P1 and P2 were set as [40, 50, 60, 70, 80, 80, 80, 80] and [40-1, 50-2, 60-2, 70-3, 80-3, 80-3, 80-3, 80-3], respectively.

**Figure 20:**
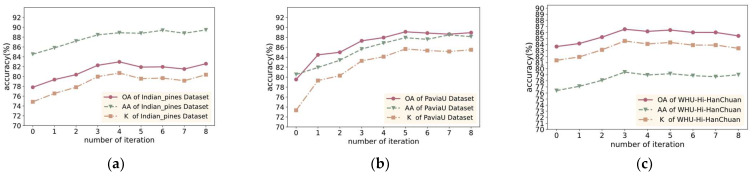
Effect of the number of DSPAL iterations on accuracy: (**a**) IP dataset; (**b**) PU dataset; (**c**) WHU dataset.

The changes in three metrics during multiple iterations on the three datasets are shown in Figure 20a–c. It can be observed that the model’s accuracy gradually improved during the iterations and reached its peak at around the fourth or fifth iteration, after which it started to fluctuate. DSPAL improved OA from 77.85% to 82.44% on the IP dataset, from 79.43% to 88.95% on the PU dataset, and from 83.31% to 86.16% on the WHU dataset. Notably, the first four iterations led to a significant improvement in OA, AA, and Kappa. On the IP dataset, the OA reached its maximum value in the fourth iteration, while on the PU and WHU datasets, the OA reached its maximum in the fifth iteration. As the number of iterations increased, the differences in OA gradually decreased. After the fifth iteration, the OA did not continue to rise but showed a decline. This is because, after a certain number of iterations, it becomes challenging to find samples with new feature information from the remaining test set. This indicates that DSPAL can find samples with new features through iteration, which significantly improves the model. However, with the decrease in new feature Information in the selected samples, too many iterations can lead to overfitting, resulting in a decrease in accuracy.

The results demonstrate that samples selected through DSPAL iterations have new features, which contribute to higher network accuracy. However, blindly increasing the number of iterations does not necessarily continuously improve the classification ability of the network. The network reaches an optimal state within a certain number of iterations, after which the network accuracy shows little improvement. Therefore, based on experimental results, this study selected four iterations of pseudo-active learning to achieve effective accuracy improvements within an appropriate range.

## 6. Conclusions

The core issues of HSI classification are accuracy and cost. The main method for reducing costs is to limit the number of annotated samples. This study proposes a multi-strategy approach to address the small-sample HSI classification problem. On the one hand, it improves the quality of annotated samples through active learning by introducing FMAL to identify valuable samples precisely. It takes into consideration the impact of the initial model accuracy on the quality of identified samples. FMAL creates anchor values from the encoding-layer features of the original training set samples. By predicting the values of unlabeled samples mixed with a certain proportion of anchor values, the value of the samples can be determined. This effectively reduces the dependence of sample selection on the original model. An efficient implementation of the optimal solution for calculating the mixed proportional weights of features is given. On the other hand, the sample size of the training set is increased through contrast learning and pseudo-active learning. To establish a reliable initial model, we introduce triplet sample pairs to expand the training samples. On this basis, DSPAL was designed to generate pseudo-label samples. This method uses two strategies to evaluate the confidence and uncertainty of the samples separately. A large number of pseudo-label samples were filtered out to expand the training set. This may be considered a promising direction for solving small-sample classification problems. Comparative experiments on three datasets demonstrated that the MSTNC method outperforms several state-of-the-art methods in terms of accuracy.

Although we have obtained encouraging experimental results, we will continue our research, specifically focusing on the following aspects:

(1) The number of samples selected in each iteration is equal to the number of samples in the training set prior to that iteration in the feature-mixture-based active learning method employed in this study. The threshold ε, which controls the feature mixing ratio, and the model’s loss in predicting the mixed feature class are two important factors in determining the quality of the selected samples. We can consider using these two values to calculate the number of selected samples in each iteration scientifically and improve the active learning method. (2) The two thresholds of the filter in pseudo-active learning were selected based on extensive experimental evaluations, and future research should aim to identify more scientific and efficient methods. (3) Our proposed dual-strategy pseudo-active method selects samples based on the classification results of the model, allowing for flexibility in the model’s structure and making it applicable to classification tasks in other fields.

## Figures and Tables

**Table 1:** Classification results of each method in Indian Pines.

Number/Class	3DCNN [20]	DFSL-NN [36]	DFSL-SVM [36]	Gia-CFSL [38]	DBDAFSL [59]	CapsGLOM [60]	MSTNC (Ours)
1. Alfalfa	90.11	98.23	97.56	**100**	**100**	**100**	**100**
2. Corn-notill	58.56	49.24	52.93	68.37	45.2	41.2	**96.76**
3. Corn-mintill	43.42	62.96	69.21	66.33	61.62	65.62	**77.91**
4. Corn	44.98	53.77	57.31	87.01	83.23	79.23	**88.05**
5. Grass-pasture	67.88	84.89	84.71	71.75	76.72	**86.72**	69.21
6. Grass-trees	89.42	92.38	92.56	84.72	92.55	**93.55**	86.63
7. Grass-pasture-mowed	91.92	98.01	98.65	99.45	**100**	**100**	**100**
8. Hay-windrowed	81.22	85.59	85.59	98.73	99.22	**100**	99.78
9. Oats	98.11	90.9	90.9	99.71	98.66	84.21	**100**
10. Soybean-notill	66.93	65.66	30.21	71.21	62.57	26.57	**73.34**
11. Soybean-mintill	19.67	73.64	95.78	44.78	70.77	**85.77**	73.71
12. Soybean-clean	30.73	21.08	39.96	54.96	38.02	**89.02**	79.06
13. Wheat	92.67	75.26	81.63	89.63	97.44	**100**	92.85
14. Woods	89.37	90.81	89.87	76.87	84.59	**93.59**	84.92
15. Buildings-Grass-Trees-Drives	55.1	58.43	40.42	80.42	88.18	78.18	**100**
16. Stone-Steel-Towers	95.06	96.53	97.8	99.02	93.52	81.52	**100**
OA	54.75 ± 3.53	65.90 ± 4.64	66.49 ± 3.37	67.52 ± 3.79	70.52 ± 3.62	74.63 ± 3.62	**82.97 ±** 2.44
AA	63.91 ± 1.45	71.25 ± 3.34	70.34 ± 2.51	80.94 ± 1.78	80.97 ± 1.77	81.58 ± 1.77	**88.89** ± 1.51
Kappa	52.21 ± 3.71	60.63 ± 4.17	60.09 ± 3.28	63.67 ± 3.92	66.67 ± 2.89	70.60 ± 2.89	**80.70** ± 2.48

**Table 2:** Classification results of each method in Pavia University.

Number/Class	3DCNN [20]	DFSL-NN [36]	DFSL-SVM [36]	Gia-CFSL [38]	DBDAFSL [59]	CapsGLOM [60]	MSTNC (Ours)
1. Asphalt	80.94	75.63	47.22	84.01	80.01	79.51	**85.06**
2. Meadows	90.56	83.78	85.31	84.83	81.83	**95.22**	92.74
3. Gravel	36.61	51.41	83.25	54.98	**92.98**	35.31	79.44
4. Trees	61.20	74.22	66.96	86.99	65.99	**94.87**	71.06
5. Painted metal sheets	99.69	98.15	99.83	**100.00**	99.77	**100.00**	**100.00**
6. Bitumen	55.92	85.46	85.2	73.22	**99.22**	60.79	83.85
7. Bare Soil	90.76	96.28	95.3	98.14	**99.14**	89.24	94.23
8. Self-Blocking Bricks	37.22	71.12	70.41	19.41	54.41	92.47	**88.69**
9. Shadows	81.49	94.98	34.12	62.02	90.02	**99.36**	86.62
OA	75.75 ± 5.56	80.47 ± 5.68	76.33 ± 3.86	76.79 ± 6.74	82.07 ± 6.74	85.60 ± 4.85	**87.94** ± 3.45
AA	70.49 ± 3.39	81.34 ± 3.85	75.20 ± 2.59	73.73 ± 5.63	84.90 ± 5.63	82.98 ± 2.64	**86.85** ± 2.37
Kappa	67.47 ± 6.45	74.64 ± 6.71	69.55 ± 4.77	69.73 ± 4.59	77.02 ± 4.59	80.76 ± 5.69	**84.11** ± 4.22

**Table 3:** Classification results of each method in WHU-Hi-HanChuan.

Number/Class	3DCNN [20]	DFSL-NN [36]	DFSL-SVM [36]	Gia-CFSL [38]	DBDAFSL [59]	CapsGLOM [60]	MSTNC (Ours)
1. Strawberry	85.97	68.26	55.16	51.52	57.36	71.22	**91.25**
2. Cowpea	80.89	45.93	46.96	67.86	58.85	76.45	**83.07**
3. Soybean	42.70	86.21	85.5	79.62	78.00	77.45	**86.90**
4. Sorghum	88.92	96.31	96.85	95.74	**97.35**	97.33	97.36
5. Water spinach	30.80	86.71	99.84	89.81	96.99	**100.00**	98.58
6. Watermelon	0.11	59.93	66.97	**84.73**	48.50	74.55	81.76
7. Greens	2.55	81.02	**98.71**	95.38	82.49	79.94	83.94
8. Trees	44.75	61.29	35.29	59.14	31.21	65.98	**76.21**
9. Grass	11.35	56.32	26.96	53.61	62.21	63.20	**83.70**
10. Red roof	39.77	66.53	73.74	61.03	**91.39**	81.54	84.47
11. Gray roof	0.04	87.74	67.8	65.52	79.59	89.06	**96.75**
12. Plastic	34.34	43.86	59.24	70.56	**74.70**	73.57	51.94
13. Bare soil	13.34	39.19	48.63	36.20	36.44	41.22	**55.11**
14. Road	24.37	65.21	34.46	30.51	52.45	57.59	**80.80**
15. Bright object	**68.10**	53.08	65.68	57.24	67.99	58.21	57.07
16. Water	98.06	72.67	98.1	96.39	**98.97**	97.72	96.48
OA	62.91 ± 2.21	67.85 ± 2.81	68.04 ± 1.65	70.15 ± 1.85	72.22 ± 2.71	79.50 ± 2.67	**86.57** ± 1.17
AA	41.71 ± 1.75	67.05 ± 2.65	66.08 ± 2.38	68.51 ± 1.78	69.58 ± 2.14	75.60 ± 1.65	**79.18** ± 1.05
Kappa	55.30 ± 2.71	63.53 ± 2.31	63.00 ± 2.42	65.67 ± 1.56	67.97 ± 2.31	76.22 ± 2.51	**84.36** ± 1.13

**Table 4:** Quantitative results for the three data sets.

DATASET	Measure	SSRN [31]	3DAES [47]	ConGcn [33]	TensorSSA [22]	SmaT-HOSVD [23]	MSTNC (Ours)
IP	OA (%)	76.16	78.93	79.94	87.55	92.45	**96.36**
	AA (%)	82.25	78.25	78.42	88.92	91.89	**96.29**
	κ × 100	72.49	75.26	77.85	86.68	93.01	**95.85**
PU	OA (%)	91.27	92.76	91.18	94.48	96.88	**100**
	AA (%)	88.50	88.50	86.50	93.45	94.45	**100**
	κ × 100	88.37	92.60	90.60	92.52	95.79	**100**
WHU	OA (%)	96.21	95.54	95.04	96.78	97.88	**100**
	AA (%)	97.21	97.91	96.01	98.02	99.01	**100**
	κ × 100	92.59	92.61	90.55	96.24	97.95	**100**

## Data Availability

The Indian Pines and Pavia University datasets are available at http://www.ehu.eus/ccwintco/index.php?title=Hyperspectral_Remote_Sensing_Scenes (accessed on 20 January 2023). The Wuhan Han Chuan dataset is available at http://rsidea.whu.edu.cn/resource_WHUHi_sharing.htm (accessed on 20 January 2023).

## Nomenclatures and Notations

MSTNC	Multi-strategy triplet network classifier
HSI	Hyperspectral Imaging
CNN	Convolutional neural networks
conv3D	3-dimensional convolutional neural networks
conv2D	2-dimensional convolutional neural networks
PCA	Principal component analysis
TN	Triplet network
TNC	Triplet networfk classifier
AL	Active learning
M	The total number of samples that can be chosen
FMAL	Feature mixture-based active learning
DSPAL	Dual- strategy pseudo-active learning
FC	Fully connected
GAP	Global average pooling layer
BvSB	Best vs. Second Best
Cls	The classes of land cover
$f_{e}$	Encoder
$f_{p}$	Projection head
$f_{c}$	Classifier
$x^{l}$	Labeled sample
$y_{i}$	Label of the i-th sample
$x^{u}$	Unlabeled sample
$D^{test}$	Test set
$D^{train}$	Train set
$x^{l+}$	The same class as $x^{l}$ (positive pair)
$x^{l-}$	The different class as $x^{l}$ (negative pair)
z	The output vector corresponding to $x^{l}$
$z^{+}$	The output vector corresponding to $x^{l+}$
$z^{-}$	The output vector corresponding to $x^{l-}$
$y_{i}$	The value of the i-th class
${\hat{y}}_{i}$	Projected value
$\theta_{p}$	Projection head parameters
$\theta_{c}$	Classifier parameters
$\bar{z}$	Anchor value
$z^{u}$	Encoding-layer feature
${\bar{z}}_{i}$	The anchor value of the i-th class of samples
${\tilde{z}}_{i}$	Mixed feature value
